# Hand eczema symptoms, exposures and skin care in orthodontics: A national, cross-sectional questionnaire-based survey

**DOI:** 10.1007/s00056-024-00524-3

**Published:** 2024-04-03

**Authors:** Bernhard Wiechens, Philipp Meyer-Marcotty, Timo Buhl, Thomas Werfel, Andrea Bauer, Christian Apfelbacher, Susann Forkel, Moritz M. Hollstein, Stephan Traidl

**Affiliations:** 1 Department of Orthodontics, University Medical Center Goettingen, Robert-Koch-Str. 40, 37075 Goettingen, Germany; 2 Department of Dermatology, Venereology and Allergology, University Medical Center Göttingen, Göttingen, Germany; 3 Lower Saxony Institute of Occupational Dermatology, University of Göttingen, Göttingen, Germany; 4 Department of Dermatology and Allergy, Hannover Medical School, Hannover, Germany; 5 Department of Dermatology, University Allergy Center, University Hospital Carl Gustav Carus, Technical University Dresden, Dresden, Germany; 6 Institute of Social Medicine and Health Systems Research, Otto von Guericke University of Magdeburg, Magdeburg, Germany

**Keywords:** Contact allergy, Hand hygiene, Epidemiology, Occupational dermatitis, Skin protection, Kontaktallergie, Handhygiene, Epidemiologie, Beruflich bedingte Dermatitis, Hautschutz

## Abstract

**Objectives:**

Occupational hand eczema is a common inflammatory skin condition among healthcare professionals. Orthodontists are frequently exposed to a variety of irritating and allergenic substances, and therefore they belong to a predisposed group to develop hand eczema. However, current data on the prevalence and predisposing factors among orthodontists to provide adequate prophylaxis are lacking.

**Methods:**

An anonymous online survey was conducted in Germany between January and February 2023 and distributed to 2402 orthodontists. The questionnaire addressed general information on current skin status, as well as occupational skin exposure and skin care.

**Results:**

A total of 209 orthodontists responded to the survey. Seventy-four percent reported experiencing hand eczema-specific symptoms within the last 12 months, with 24% describing moderate and 10% describing severe symptoms. The average daily glove wearing time was stated to be 6 ± 2 h. The most frequently reported triggers at work were frequent hand washing (62.7%) and hand disinfection (59.1%). Among all the respondents, 22.6% stated not using either barrier cream or moisturizer.

**Conclusions:**

This study showed a high prevalence of hand eczema symptoms among orthodontists, which is probably due to frequent disinfection, hand washing, and contact with allergens such as acrylates. In this professional group especially, against a background of future increasing acrylate and epoxy resin exposures due to in-office three-dimensional printing processes, timely education and skin protection could decisively counteract the pathogenesis of hand eczema.

**Supplementary Information:**

The online version of this article (10.1007/s00056-024-00524-3) contains supplementary material, which is available to authorized users.

## Abstract (de)

**Zielsetzung:**

Das berufsbedingte Handekzem ist eine häufige entzündliche Hauterkrankung bei Heilberufstätigen. Kieferorthopäd*innen sind häufig einer Vielzahl von reizenden und allergenen Substanzen ausgesetzt und gehören daher zu einer prädisponierten Gruppe für die Entwicklung eines Handekzems. Es fehlen jedoch aktuelle Daten über die Prävalenz und prädisponierende Faktoren bei Kieferorthopäd*innen, um eine angemessene Prophylaxe zu gewährleisten.

**Methoden:**

Eine anonyme Online-Umfrage wurde zwischen Januar und Februar 2023 in Deutschland durchgeführt und an 2402 Kieferorthopäden verschickt. Der Fragebogen adressierte allgemeine Informationen zum aktuellen Hautstatus sowie zu beruflich bedingter Hautexposition und Hautpflege.

**Ergebnisse:**

Insgesamt 209 Kieferorthopäd*innen nahmen an der Umfrage teil. Vierundsiebzig Prozent gaben an, in den letzten 12 Monaten an handekzemspezifischen Symptomen gelitten zu haben, wobei 24% moderate und 10% schwere Symptome beschrieben. Die durchschnittliche tägliche Tragezeit von Handschuhen wurde mit 6 ± 2 h angegeben. Die am häufigsten genannten Auslöser bei der Arbeit waren häufiges Händewaschen (62,7%) und Händedesinfektion (59,1%). Von allen Teilnehmenden gaben 22,6% an, weder Hautschutz- noch Feuchtigkeitscremes zu verwenden.

**Schlussfolgerung:**

Die Studie ließ eine hohe Prävalenz von Handekzemsymptomen bei Kieferorthopäd*innen beobachten, was wahrscheinlich auf häufiges Desinfizieren und Händewaschen sowie den Kontakt mit Allergenen, wie Acrylaten, zurückzuführen ist. Vor dem Hintergrund der zukünftig zunehmenden Acrylat- und Epoxidharzbelastung durch 3‑D-Druckverfahren in der Praxis könnte gerade in dieser Berufsgruppe durch rechtzeitige Aufklärung und Hautschutz der Pathogenese des Handekzems entscheidend entgegengewirkt werden.

## Introduction

Hand eczema is an inflammatory condition characterized by symptoms such as redness, scaling, and painful fissures [3]. Occupational hand eczema is frequently suspected and diagnosed in Germany, often leading to long periods of inability to work [14]. Hand eczema may impair health-related quality of life in a way similar to other chronic conditions such as cancer or hepatitis [8]. Its prevalence is much higher among healthcare professionals (up to 74.5% [29]) than among people in other occupations (lifetime prevalence in the general population: 14.5% [19, 30, 36]). Since intensive and frequent hand hygiene are major risk factors [30], some studies suggest the highest risk of developing hand eczema across all occupational groups in dentistry [24, 26, 40]. Correspondingly, the risk of developing hand dermatitis and eczema has been found to be higher among dental personnel than in the general population [26]. Further, the prevalence of hand eczema even differs between dental specialties [26]. Symptoms are reported in 17–28% of dental laboratory personnel and in up to 40–43% of professionals involved in dental treatment, with women affected 1.5–2 times more frequently than men [15, 16, 18, 23, 25, 37, 43]. Among the main causes of hand eczema-related symptoms, specifically among healthcare professionals, are wet work, frequent hand washing and disinfection [40], allergies to latex gloves or vulcanization accelerators [6, 35], dental composites, bonding agents, and accessory stabilizers with chemically active intermediates [20, 38]. In this context, (meth)acrylates are major chemically active intermediates that cause reactions on hands and fingertips [28, 38, 46, 47].

Previous studies have provided solid data on occupation-specific differences in skin exposure. For example, the prevalence of self-reported hand dermatitis symptoms was higher among orthodontists and their assistants (50.4%) than among periodontists or prosthodontists (42%) [12, 15–17]. For the different work domains, such as dental assistants and dental technicians, prevalences of hand-eczema symptoms have already been reported (36% [4] and 35% [33]). At the same time, the high number of patient cases, especially in orthodontics, necessitates frequent glove changes or hand washing and disinfection routines, which also likely contribute to the increased hand eczema prevalence in orthodontists [32]. Finally, the increasing importance of computer-aided design and computer-aided manufacturing (CAD-CAM) approaches in orthodontics may translate into an increased hand eczema incidence in the field [41]. In addition to metal printing, which was recently developed [10], acrylic-based three-dimensional (3D) printing of models and aligners is becoming increasingly common in practice [41]. This tremendous impact can be directly observed in the United States, the world’s largest market for dental 3D printing applications, where the orthodontics segment accounted for the largest market share compared to prosthodontics and implantology, with 39.0% of total revenue in 2022, which is expected to grow substantially according to current forecasts [1]. This explosive growth of 3D printing can also be observed in Germany and will undoubtedly lead to a generally higher and more frequent exposure to acrylates in the unpolymerized state given the frequently observed in-office applications. According to a recently published ex vivo study, the toxicity of (meth)acrylates appear to be far more extensive than previously assumed [39]. This may translate into an increasing hand eczema disease burden. The purpose of this study was to estimate the prevalence of hand eczema symptoms among orthodontists and identify preventable risk factors to enable future preventive measures.

## Methods

The present cross-sectional study was conducted in Germany in January and February 2023 and was based on an anonymous online questionnaire. The questionnaire was sent by e‑mail to publicly available email addresses of 2402 orthodontists, which covered more than 64.4% of orthodontists in Germany (3731 total, 2895 ambulatory) [7]. Two electronic reminders were sent at intervals of 2 weeks. In addition, the questionnaire was distributed via the Federation of German Orthodontists (Bund Deutscher Kieferorthopäden, Berlin, Germany). Duplicated submissions of the questionnaire were conceivable in principle, but unlikely due to the extent of the questionnaire. Participants were included if they provided written informed consent and specified orthodontics as their primary profession. We excluded all other orthodontic practice personnel. Evasys survey software (version 9.1, evasys GmbH, Lüneburg, Germany) was used to design the questionnaire (Supplementary Fig. 1) and capture the data.

The questionnaire covered relevant aspects regarding allergen exposure and skin care. We divided the 63 questions into 4 groups: (1) general information including age, sex, smoking status, and nonoccupational skin factors, (2) current skin status, (3) occupational skin stress including exposure to allergens and irritants, and (4) skin care (see questionnaire in the supplementary figure 1). Unfinished questionnaires were not excluded from the analysis and individual questions could be left unanswered.

This study was conducted in accordance with the principles of the Declaration of Helsinki and was approved by the ethics committees of Hannover Medical School (No. 10357_B0_K_2022). It was registered with the German Clinical Trials Register prior to the start of the study (DRKS00026677) and complies with the Consensus-Based Checklist for Reporting of Survey Studies (CROSS) [42]. All participants provided written consent for the anonymous evaluation of information and the exchange of data between the participating university hospitals.

## Statistical analysis

R (version 4.1.2, The R Foundation for Statistical Computing, Vienna, Austria) was used for analysis of the survey data. In particular, the gtsummary package (version 1.7.0) was used. The results are presented using descriptive statistics. For metric variables, calculations were performed for the means and standard deviations, as well as medians and interquartile ranges. Nominal variables are displayed using relative and absolute frequencies. Figures were created using GraphPad Prism (version 9.5.1, GraphPad Software, Boston, MA, USA). The absolute number of responses (i.e., nonmissing values) is indicated for each item in the respective figures and tables. Relative frequencies are based on the number of responses given. There was no imputation of missing responses.

## Results

### Sociodemographic data

Among the 2402 orthodontists contacted by January 5, 2023, 253 questionnaires were not delivered due to restrictive firewall settings, resulting in a net distribution of 2149 questionnaires. After two reminders at 2‑week intervals and a parallel notification in the newsletter of the Federation of German Orthodontists (Bund Deutscher Kieferorthopäden, Berlin, Germany) on January 24, 2023, 209 questionnaires could be evaluated after completion of the survey, representing a response rate of 9.7%. Eighty-two respondents were male (39.4%) and 126 (60.6%) were female orthodontists with an average age of 45.9 ± 11.3 years. In 78.5% of cases, they were nonsmokers, and in 85.2% of cases, they were employed full-time with a median of 20 years of professional experience. The average daily time share spent on clinical versus office activities was answered on a visual analogue scale. Participating orthodontists indicated that 58.9% of the time in a typical working day was spent on clinical activities. Secondary occupations were reported by 4.8% of the respondents (Table 1).

**Table 1:** Demographic and employment characteristics, including means and standard deviations (SD) as well as medians and interquartile ranges (IQR) Demographische und beschäftigungsbezogene Merkmale einschließlich Mittelwerten und Standardabweichungen (SD) sowie Medianen und Interquartilbereichen (IQR)

*Age (years)*	*n* *=* *200, (95.7%)*
Mean (SD)	45.9 (11.3)
Median (IQR)	47.0 (18.3)
*Sex*	*n* *=* *208, (99.5%)*
Male	82 (39.4%)
Female	126 (60.6%)
*Smoking status*	*n* *=* *209, (100%)*
Unknown	3 (1.4%)
Never	164 (78.5%)
Stopped over 10 years ago	21 (10.0%)
Stopped in the last 10 years	13 (6.2%)
Current	8 (3.8%)
*Employment status*	*n* *=* *209, (100%)*
Part-time or hourly	31 (14.8%)
Full-time (38 h or more)	178 (85.2%)
*Workshare in medical activities (vs office activities) (%)*	*n* *=* *209, (100%)*
Mean (SD)	58.9 (18.6)
Median (IQR)	60.0 (20.0)
*Second employment (%)*	*n* *=* *208, (99.5%)*
No	198 (95.2%)
Yes	10 (4.8%)
*Number of years in current employment (years)*	*n* *=* *208, (99.5%)*
Mean (SD)	19.5 (11.0)
Median (IQR)	20.0 (19.0)

### Experience with eczema-specific skin symptoms

**Fig. 1:**
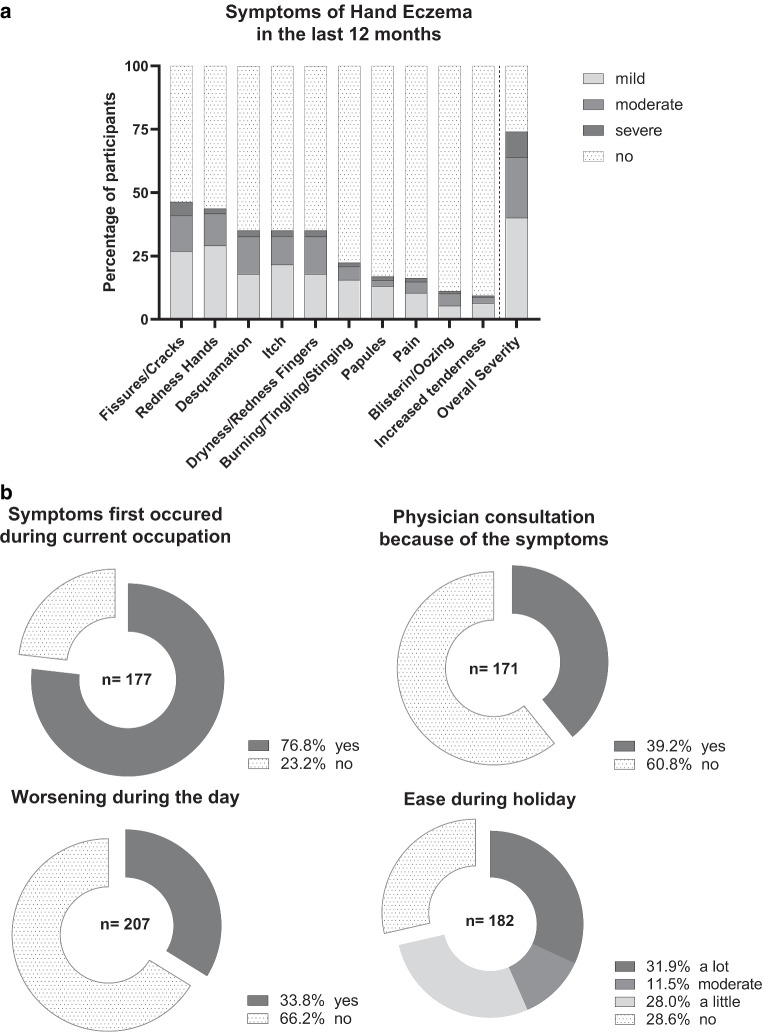
**a** Symptoms of hand eczema in the last 12 months. *Stacked bars* illustrate the distribution of specific symptoms in percentages based on four severity levels among all participants within the past 12 months (*n* = 207; 99% of participants). **b** Eczema-specific symptoms. Pie charts illustrate the dichotomous statement regarding symptom-specific questions (in %) with special consideration for varying numbers of participants (*n* = 207; 99% of participants) **a** Handekzemsymptome in den letzten 12 Monaten. *Übereinanderliegende Balken* veranschaulichen die prozentuale Verteilung spezifischer Symptome auf Grundlage von 4 Schweregraden unter allen Teilnehmenden innerhalb der letzten 12 Monate (*n* = 207; 99 % der Teilnehmenden). **b** Ekzemspezifische Symptome. Kreisdiagramme veranschaulichen die dichotomen Aussagen zu symptomspezifischen Fragen in (%) unter besonderer Berücksichtigung der unterschiedlichen Teilnehmendenzahlen (*n* = 207; 99 % der Teilnehmenden)

The participants were surveyed for a self-assessment of eczema-specific skin symptoms and individual morphological patterns (Fig. 1a). In this regard, Fig. 1a illustrates a detailed symptom distribution of all respondents according to descending frequency and shows the absolute distribution of eczema-related symptoms across all respondents. Three in four orthodontists (74%) experienced hand eczema-specific symptoms in the last 12 months. Regarding the overall severity of symptoms, 24% of participants reported moderate and 10% reported severe symptoms. In addition, 14.1% of the participants reported moderate fissures and cracks, while 5.4% reported severe fissures and cracks. Moreover, 4.4% of respondents reported moderate pain, and 1.5% reported severe pain. Furthermore, 2.5% suffered from moderately increased tenderness, while 0.5% suffered from severely increased tenderness. Figure 1b further reveals that in more than 76.8% of cases, symptoms first occurred in the current occupation and appeared to be work related, being worse during the day (33.8%) and subsiding during vacations (71.4%).

The eczema-focused medical histories of the respondents (Table 2, Supplementary Table 1) revealed that moderate skin dryness was present in 51.7% and severe skin dryness in 22.0% of corresponding cases. Although 82.7% of respondents reported no history of suspected contact dermatitis, i.e., allergic sensitization to specific allergens, 21.3% reported having a diagnosed eczema, of which 23.9% reported the manifestation of this eczema on both hands and/or forearms. The most frequently reported triggers at work were frequent hand washing (62.7%) and hand disinfection (59.1%; Supplementary Table 2).

**Table 2:** Eczema-focused medical history, showing number (*n*) and percentage in the corresponding subgroup Ekzemspezifische Anamnese mit Anzahl (*n*) und Prozentsatz in der entsprechenden Untergruppe

*History of dry skin (%)*	*n* *=* *209, (100%)*
Unknown	1 (0.5%)
No	54 (25.8%)
Yes, somewhat	108 (51.7%)
Yes, very	46 (22.0%)
*History of suspected contact dermatitis (%)*	*n* *=* *208, (99.5%)*
No	172 (82.7%)
Yes	36 (17.3%)
*Physician-diagnosed eczema (%)*	*n* *=* *207, (99.0%)*
Unknown	8 (3.9%)
No	155 (74.9%)
Mild	18 (8.7%)
Moderate	13 (6.3%)
Strong	13 (6.3%)
*➥ Diagnosed eczema was located on forearms (%)*	*n* *=* *46*
No	35 (76.1%)
Yes	11 (23.9%)

➥ indicates the proportion for the topic of the previously mentioned corresponding subgroup

### Workplace factors and impairment

With regard to workplace factors and impairment related to skin health (Table 3, Supplementary Table 3), respondents reported an average daily time of glove wearing of 6.0 ± 2.0 h. Orthodontists reported on average 14.0 ± 14.4 daily hand washing routines and 28.8 ± 21.0 daily disinfection routines. Only one respondent reported that hand-related skin problems resulted in the inability to work (for 2 months). Employment-related preventive skin protection measures were regularly available in 25.5% of orthodontists’ work environments and were reported to be absent by 39.9%. Most respondents (93.8%) commented on the influence of materials, chemicals, or other factors on their hand eczema-related symptoms. More than half (50.5%) reported no effect on the skin, while 37.8% confirmed a flare-up caused by these agents. Of those, 39 respondents commented on the time lapse until exacerbation after contact, which was, on average, 2.9 ± 4.2 h. Among all respondents, 53.1% reported worsening of hand eczema-related symptoms during wintertime.

**Table 3:** Workplace factors and impairment with number (*n*) of responses and percentages Arbeitsplatzbezogene Faktoren und Beeinträchtigungen mit Anzahl (*n*) der Antworten und Prozentsätzen

*Hours gloves are worn at work*	*n* *=* *201, (96.2%)*
Mean (SD)	6.1 (2.0)
Median (IQR)	6.0 (2.0)
*Handwashing procedures [Frequency/day]*	*n* *=* *185, (88.5%)*
Mean (SD)	14.0 (14.4)
Median (IQR)	8.0 (15.0)
*Hand disinfection [Frequency/day]*	*n* *=* *187, (89.5%)*
Mean (SD)	28.8 (21.0)
Median (IQR)	25.0 (30.0)
*Months out of work due to skin problems [months]*	*n* *=* *187, (89.5%)*
Mean (SD)	0.0 (0.2)
Median (IQR)	0.0 (0.0)
*Employment-related eczema prevention measures*	*n* *=* *208, (99.5%)*
Never	83 (39.9%)
Sometimes	72 (34.6%)
Regularly	53 (25.5%)
*Exacerbation through materials, chemicals, or other factors*	*n* *=* *196, (93.8%)*
No	99 (50.5%)
Unclear	23 (11.7%)
Yes	74 (37.8%)
*➥Time lapse until exacerbation after contact [hours]*	*n* *=* *39*
Mean (SD)	2.9 (4.2)
Median (IQR)	1.0 (2.0)
*Exacerbation in winter*	*n* *=* *209, (100%)*
No	98 (46.9%)
Yes	111 (53.1%)

*SD* standard deviation, *IQR* interquartile range

➥ indicates the proportion for the topic of the previously mentioned corresponding subgroup

### Skin cream use

**Fig. 2:**
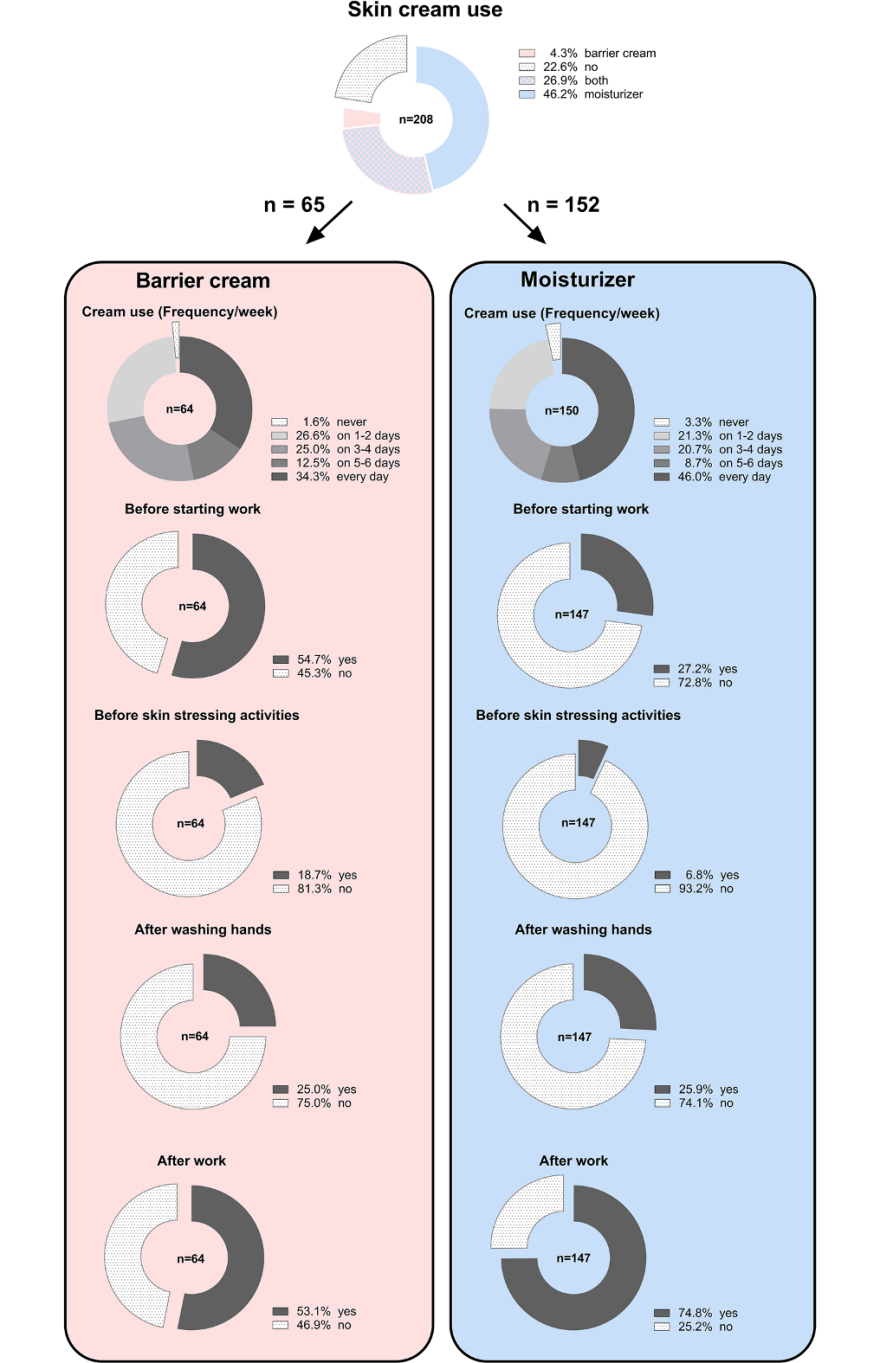
Skin cream use. Pie charts showing the overall distribution and subdivision of various aspects of skin cream use in (%) with special consideration for varying numbers of participants (*n*). *Blue *participants who used skin moisturizer, *red *participants who used barrier cream, and *red–blue* who used both skin moisturizer and skin barrier cream Hautcremeanwendung. Kreisdiagramme zur Gesamtverteilung und Unterteilung verschiedener Aspekte der Hautcremeanwendung in (%) unter besonderer Berücksichtigung der unterschiedlichen Teilnehmerzahlen (*n*). *Blau *bezieht sich auf die Teilnehmenden, die Feuchtigkeitscreme verwendeten. *Rot *bezieht sich auf die Teilnehmenden, die Schutzcreme verwendeten. *Rot-blau *gibt den Anteil der Teilnehmenden an, die sowohl Feuchtigkeits- als auch Schutzcreme verwendeten

Almost all respondents (*n* = 208) answered the question of whether they used a skin moisturizer, barrier cream, none, or both. In total, 22.6% stated that they used neither barrier cream nor moisturizer (Fig. 2). In contrast, 26.9% of the respondents, and thus only slightly more, stated that they used both barrier cream and moisturizer. Just under half of all respondents (46.2%) solely used a moisturizer.

Barrier cream alone was used by 4.3% of participating orthodontists. With regard to the frequency of application, one-third of barrier cream users stated that they used it daily (34.3%), 54.7% used it before and 53.1% used it after work. An additional 25.0% used it after washing their hands. Interestingly, we registered 1.6% of participants who reported using barrier cream but who also responded “never” to the frequency of barrier cream use per week. For moisturizers, this finding applied to 3.3% of respondents.

Among the users of moisturizers, just under half of the respondents (46.0%) stated that they used moisturizers daily. Just about one-quarter of users applied moisturizer before (27.2%) and three quarters of users (74.8%) applied moisturizer after work. Almost no responder used a moisturizer before skin stress activities (6.8%), whereas 25.9% applied it after washing their hands.

## Discussion

In the present study, 74% of the respondents reported specific hand eczema-related skin lesions within the past 12 months. This indicates a significantly higher prevalence than that reported by Jacobson and Hensten-Pettersen in 1989 [16]. They reported a frequency of hand eczema among 40% of 137 Norwegian orthodontists surveyed. Among 3500 Swedish dentists, Wallenhammer et al. found a prevalence of 14.9% of self-reported hand eczema within the past 12 months [46]. Considering the 1‑year prevalence in the general population, a meta-analysis of seven Scandinavian and Dutch studies that included a total of 16,754 study participants showed a prevalence of 9.1% (95% confidence interval [CI] 8.9–9.3) [44].

Overall, the present study demonstrated a high prevalence of hand eczema symptoms among orthodontists compared to the general population and other dental health professionals. Possible differences in the survey methodology may contribute to these findings. In addition, response bias may have influenced our results, as orthodontists with hand eczema may have been more likely to respond. The response rate was 9.7% of 2149 distributed questionnaires. The generalizability of our results may be limited considering that there are slightly more than 3700 orthodontists in Germany [7]. Another possible drawback of this study is its nonvalidated questionnaire, which we were forced to develop due to the lack of a validated questionnaire addressing the study question. Further data are needed to evaluate whether there has been an increase over time or if this is a study-specific finding, as there are no current data from other dental professions.

An average of 13.9 hand washing procedures and 28.8 disinfections per day were reported in this study, showing frequent exposure to skin irritations. Lund et al. found in their study among 3333 men and 11,908 women who performed wet work, including healthcare workers, that even with an average exposure of ≤ 30 min of wet work per week, there was an increased risk of hand eczema [31]. Wearing gloves was associated with an increased risk of hand eczema among women but not among men. However, studies have also demonstrated the protective effects of gloves [22]. Following the recommendation of the current European guidelines on hand eczema, wearing gloves is recommended for orthodontists for hand eczema prevention [45]. Considering the glove wearing time of 6 ± 2 h in the present study, this protective measure seems to be well implemented.

Upon surveying orthodontists, the most frequently reported exacerbating factors were hand washing (63%) and hand disinfection (59%), which suggest irritant occupational hand eczema. This is consistent with the findings by Wallenhammer et al. [46], who found a higher prevalence of irritant contact dermatitis compared to allergic contact dermatitis among 3500 dentists aged < 65 years (67% vs. 28%), and is further supported in a recently published review [20]. In addition, 29% of orthodontists in the present study reported a worsening of symptoms due to work-related stress. Accordingly, Japundžić et al. [21] observed in a study of 148 physicians and dentists that high stress levels were associated with 2.5 higher odds for self-reported hand eczema. Conversely, stress levels were lower in those who did not report hand eczema [21]. Recently published data on 1491 patients with occupational hand eczema showed that the prognosis of the condition was significantly influenced by smoking and stress, while contact sensitization was not a negative predictor [34]. Notably, the rate of active smokers was significantly lower (3.8%) in the present study than in the general population, which was reported to be approximately 23.8% by the German Federal Ministry of Health in 2021 [2].

Many participants (38%) described a worsening of the skin condition following the use of specific materials and agents. These exacerbations could have been caused by so far unrecognized contact allergies. However, symptom aggravation within a few hours after contact suggests an irritative etiology rather than an underlying contact sensitization. In orthodontics, a variety of potential contact allergens are regularly used, including acrylates and methacrylates [9]. Patch test data from the Information Network of Departments of Dermatology (IVDK) from 2001–2015 showed that acrylates and methacrylates were the predominant allergens among dental technicians, with 67 out of 226 (29.6%) showing a sensitization [13]. These substances are increasingly used, especially in 3D printing. It should be noted that nitrile gloves, which are typically worn, do not provide protection against acrylates and methacrylates for more than 10 min [11]. A structured monitoring program with organizations such as the IVDK is needed to evaluate whether sensitizations among orthodontists will increase in the future and require further intervention.

Given the high prevalence reported, adequate prevention cannot be overemphasized. In a Cochrane review on the prevention of occupational hand dermatitis by Bauer et al., nine randomized controlled studies were analyzed with 2888 individuals without occupational irritant hand dermatitis at baseline. A total of 1533 subjects received skin care through moisturizers, barrier creams, or both, and 1355 subjects were educated regarding skin protection [5]. The authors concluded that moisturizers or a combination of moisturizers and barrier creams had protective effects, but there was insufficient evidence for positive effects in patient education regarding skin protection due to a lack of proper studies. However, not only the potential protective effect is important but also the improvement of already manifested hand eczema through adequate topical therapy. Among other studies, a recently published double-center randomized study on the secondary prevention of hand contact dermatitis showed that educating patients on the use of skin protection led to a significant improvement (52.5–63%) among 102 patients with hand eczema [27]. The present study found out that more than 20% of orthodontists refrained from using either barrier cream or moisturizer, and only approximately 25% used both types of creams. Education on the benefits and potential protective effects of barrier cream and moisturizer could be a useful measure in this regard. Already established interdisciplinary concepts in cooperation with social accident insurance institutions could be a possible structure to be utilized to overcome hand eczema-related hazards. We encourage orthodontic colleagues to rethink their already implemented skin prevention measures and find further easy-to-implement measures to overcome the high burden of hand eczema-related adverse skin reactions.

## Conclusion

The present study highlights the relevance of occupational skin irritation among orthodontists. It revealed a high prevalence of hand eczema symptoms among orthodontists in Germany. Frequent disinfection and hand washing as well as exposure to allergens such as acrylate could be an explanation. Based on these findings, increased skin protection measures could be crucial for the prevention and alleviation of hand eczema among orthodontists. Especially prior to the advancing development of in-office computer-aided design and computer-aided manufacturing (CAD-CAM) approaches, which will be associated with increased exposure to (meth)acrylates, the present study results should draw attention to the great importance of skin care at the right time.

## Supplementary Information

Supplementary Fig. 1 questionnaire

Supplementary Tables 1–3
