# Supplementary material for: Hand eczema symptoms, exposures and skin care in orthodontics: A national, cross-sectional questionnaire-based survey
Source: J Orofac Orthop. 2024 Apr 3;86(5):327–36. doi: 10.1007/s00056-024-00524-3 (PMC12373531; doi:10.1007/s00056-024-00524-3)
Supplement: Supplementary file 1 — Supplementary Fig. 1 questionnaire [file 56_2024_524_MOESM1_ESM.pdf]

evasys	KFO Allergologie	evasys

DGAKI KFO - Allergologie UMG

Bitte so markieren: ☐ ☒ ☐ ☐ ☐ Bitte verwenden Sie einen Kugelschreiber oder nicht zu starken Filzstift. Dieser Fragebogen wird maschinell erfasst.
Korrektur: ☐ ■ ☐ ☒ ☐ Bitte beachten Sie im Interesse einer optimalen Datenerfassung die links gegebenen Hinweise beim Ausfüllen.

Einwilligungserklärung

Ich habe den Text der Teilnehmerinformation gelesen und hatte ausreichend Zeit, mich zu entscheiden. Ich erkläre mich damit verstanden, dass meine Daten aus diesem Fragebogen in **anonymer Form** gespeichert und ausgewertet werden. Anonym bedeutet, dass die Daten nicht meiner Person zugeordnet werden können. Die anonymen Daten werden ausgewertet durch die Mitarbeiter/innen der Arbeitsgruppe von Herrn Prof. Dr. Timo Buhl von der Universitätsmedizin Göttingen sowie Herrn Prof. Dr. Thomas Werfel, Medizinische Hochschule Hannover.
Ich erkläre mich bereit, freiwillig an dieser Studie teilzunehmen.

☐ Ja ☐ Nein

Allgemeine Informationen

Welchen Beruf üben Sie aus?
☐ Zahnärztin / Zahnarzt ☐ Zahnmedizinische/r Fachangestellte/r ☐ Sonstige

In welchem Beschäftigungsverhältnis arbeiten Sie?
☐ Vollzeit (38h oder mehr) ☐ Teilzeit oder stundenweise ☐ Auszubildende/r
☐ Praktikant/in

Wie viel Prozent Ihrer Arbeit verbringen Sie mit Büro- und medizinischen Tätigkeiten?
nur Büro ☐ ☐ ☐ ☐ ☐ ☐ ☐ ☐ ☐ ☐ ☐ nur medizinische Tätigkeit

Seit wie vielen Jahren arbeiten Sie in Ihrem jetzigen Beruf?
(keine Angabe gewünscht: Eingabe: NA)

Gehen Sie einer Nebentätigkeit nach?
☐ Nein ☐ Ja, folgende:

Geschlecht
☐ Weiblich ☐ Männlich ☐ Divers
☐ Keine Angabe

Wie alt sind Sie?
(keine Angabe gewünscht: Eingabe: NA)

Rauchen Sie oder haben Sie früher einmal geraucht?
☐ Ja, aktuell ☐ Früher, in den letzten 10 Jahren aufgehört ☐ Früher, seit über 10 Jahren nicht mehr
☐ Nein, nie ☐ Keine Angabe

MUSTER

MUSTER

Allgemeine Informationen [Fortsetzung]

Neigen Sie zu trockener Haut?	☐ Ja, sehr<br>☐ Weiß nicht	☐ Ja, etwas	☐ Nein
Bestehen außerberufliche Hautbelastungen (Gartenarbeit, Hausbau,-renovierung, Pflege Angehöriger, hautbelastende Hobbies, z.B. Töpfern)	☐ Nein	☐ Ja, folgende:	

Fragen zum aktuellen Hautzustand

Hatten Sie jemals einen juckenden Ausschlag, der über mindestens 6 Monate immer wieder auftritt und irgendwann auch die nachfolgenden Hautareale betroffen hat: Beuge der Ellenbogen, Kniekehle, an der Vorderseite der Knöchel, unterhalb vom Gesäß, um den Hals, an den Ohren oder um die Augen?	☐ Nein	☐ Ja	
Hatten Sie jemals Heuschnupfen (Nasenlaufen, Niesen, Augentränen) oder Asthma nach Kontakt mit Allergenen (z.B. Baumpollen, Tierhaare, Gräser, Hausstaubmilben)?	☐ Nein	☐ Ja, folgende:	
Bekommen sie Ausschlag von Metallknöpfen, Schmuck wie Ohrringen, Uhren oder vergleichbaren Metallgegenständen?	☐ Nein	☐ Ja	
Wurden bei Ihnen jemals ein Handekzem ärztlich diagnostiziert und falls ja, wie stark war dieses ausgeprägt?	☐ Ja, stark<br>☐ Nein	☐ Ja, mittel<br>☐ Weiß nicht	☐ Ja, mild
Betraf das Ekzem auch Handgelenke und/oder Unterarme?	☐ Nein	☐ Ja	
Wann hatten sie das Ekzem?	☐ Aktuell<br>☐ früher, vor mehr als 12 Monaten	☐ früher, in den letzten 3 Monaten	☐ früher, in den letzten 3-12 Monaten

Liegen/lagen bei Ihnen in den letzten 12 Monaten folgende Hautveränderungen bzw. Symptome an den Händen und/oder Fingern vor?

Trockene Stellen und Rötungen im Bereich der Fingerzwischenräume	☐ Nein<br>☐ Schwer	☐ Leicht	☐ Mäßig
Schuppung	☐ Nein<br>☐ Schwer	☐ Leicht	☐ Mäßig
Rötung	☐ Nein<br>☐ Schwer	☐ Leicht	☐ Mäßig

MUSTER

MUSTER

Fragen zum aktuellen Hautzustand [Fortsetzung]

Bläschenbildung / Nässen	☐ Nein ☐ Schwer	☐ Leicht	☐ Mäßig
Fissuren / Risse	☐ Nein ☐ Schwer	☐ Leicht	☐ Mäßig
Papeln / kleine Pickel	☐ Nein ☐ Schwer	☐ Leicht	☐ Mäßig
Juckreiz	☐ Nein ☐ Schwer	☐ Leicht	☐ Mäßig
Brennen / Kribbeln / stechendes Gefühl	☐ Nein ☐ Schwer	☐ Leicht	☐ Mäßig
Druckempfindlichkeit	☐ Nein ☐ Schwer	☐ Leicht	☐ Mäßig
Schmerzen	☐ Nein ☐ Schwer	☐ Leicht	☐ Mäßig

An wie vielen Monaten **der letzten 12 Monate** war Ihre Haut an den Händen und / oder Fingern aktiv betroffen?

☐☐

Wie sehr haben Sie die zuvor genannten Hautveränderungen bzw. Symptome **in den letzten 7 Tagen** belastet?
0 (überhaupt nicht belastend) ☐☐☐☐☐☐☐☐☐☐☐ 10 (extrem belastend)

Wie sehr haben Sie die zuvor genannten Hautveränderungen bzw. Symptome belastet, **als es am schlimmsten war**?
0 (überhaupt nicht belastend) ☐☐☐☐☐☐☐☐☐☐☐ 10 (extrem belastend)

Haben Sie bei Erstauftreten der angegebenen Symptome schon in ihrem jetzigen Job gearbeitet
☐ Ja ☐ Nein, sondern damals noch als (Beruf)

In welchem Lebensjahr traten die angegebenen Symptome zuerst längerfristig auf?
☐ ≤ 6. Lebensjahr ☐ 6-14. Lebensjahr ☐ 15-18. Lebensjahr ☐ ≥18. Lebensjahr

Haben Sie diese Symptome bereits ärztlich abklären lassen?
☐ Nein ☐ Ja

Verschlechtern sich die Symptome in einer bestimmten Jahreszeit?
☐ Nein, keine Abhängigkeit von Jahreszeiten ☐ Verschlechterung im Frühling ☐ Verschlechterung im Sommer
☐ Verschlechterung im Herbst ☐ Verschlechterung im Winter

Fragen zur Hautbelastung

Haben Sie festgestellt, dass der Kontakt mit gewissen Materialien, Chemikalien oder andere Faktoren das Handekzem bzw. die oben angegebenen Symptome verschlimmert?
☐ Ja ☐ Nein ☐ Unklar

MUSTER

Fragen zur Hautbelastung [Fortsetzung]

Welche Materialien/Chemikalien/Faktoren sind das bei der Arbeit?

- ☐ Handschuhe
- ☐ Reinigungsmittel, inkl. Waschmittel und Seife
- ☐ Lösungsmittel
- ☐ Desinfektionsmittel
- ☐ häufiges Händewaschen
- ☐ Arbeit mit feuchten oder nassen Händen
- ☐ Stress
- ☐ Nahrungsmittel/-zubereitung
- ☐ Öle
- ☐ Farben
- ☐ Kleber
- ☐ Staub
- ☐ Abfall, Müll
- ☐ Weiteres:

Welche Materialien/Chemikalien/Faktoren sind das in der Freizeit?

- ☐ Handschuhe
- ☐ Reinigungsmittel, inkl. Waschmittel und Seife
- ☐ Lösungsmittel
- ☐ Desinfektionsmittel
- ☐ häufiges Händewaschen
- ☐ Arbeit mit feuchten oder nassen Händen
- ☐ Stress
- ☐ Nahrungsmittel/-zubereitung
- ☐ Öle
- ☐ Farben
- ☐ Kleber
- ☐ Staub
- ☐ Abfall, Müll
- ☐ Weiteres:

Wie viele **Stunden** nach Kontakt werden die Hautveränderungen üblicherweise schlimmer?
(keine Angabe gewünscht: Eingabe: NA)

Wie viele Stunden pro Tag tragen Sie Handschuhe bei der **Arbeit**?
(keine Angabe gewünscht: Eingabe: NA)

Welche Art Handschuhe nutzen Sie bei der Arbeit?

- ☐ Latex/natürliche Gummiverbindungen
- ☐ synthetische Gummiverbindungen (u.a. Nitril, Neopren)
- ☐ Plastik (u.a. PVC bzw. Vinyl)
- ☐ Leder
- ☐ Andere
- ☐ Weiß nicht

Wie viel Mal waschen Sie die Hände **pro Arbeitstag**?
(keine Angabe gewünscht: Eingabe: NA)

Fragen zur Hautbelastung [Fortsetzung]

Wie viel Mal desinfizieren Sie die Hände **pro Arbeitstag**?
(keine Angabe gewünscht: Eingabe: NA)

An wie vielen Monaten in den letzten 12 Monaten konnten Sie aufgrund der Hautprobleme an den Händen nicht erwerbstätig sein?
(keine Angabe gewünscht: Eingabe: NA)

Fragen zum Hautschutz

Kennen Sie den Unterschied zwischen Hautschutz- und Hautpflegecreme?
☐ Ja ☐ Nein ☐ Keine Angabe

Verwenden Sie Hautschutzcreme und/oder Hautpflegecreme?
☐ Hautschutzcreme ☐ Hautpflegecreme ☐ Beides ☐ Nein

An wie vielen Tagen in der Woche verwenden Sie eine Hautschutzcreme?
☐ An keinem Tag ☐ An 1-2 Tagen ☐ An 3-4 Tagen ☐ An 5-6 Tagen ☐ An jedem Tag

Wenn mind 1-2/Tag: Wann wenden Sie die Hautschutzcreme an? (Mehrfachauswahl)
☐ Vor Arbeitsbeginn ☐ Vor hautbelastenden Tätigkeiten ☐ Nach dem Händewaschen ☐ Nach der Arbeit

An wie vielen Tagen in der Woche verwenden Sie eine Hautpflegecreme?
☐ An keinem Tag ☐ An 1-2 Tagen ☐ An 3-4 Tagen ☐ An 5-6 Tagen ☐ An jedem Tag

Wenn mind 1-2/Tag: Wann wenden Sie die Hautpflegecreme an? (Mehrfachauswahl)
☐ Vor Arbeitsbeginn ☐ Vor hautbelastenden Tätigkeiten ☐ Nach dem Händewaschen ☐ Nach der Arbeit

Gibt es an ihrer Arbeitsstelle betriebliche Präventionsschulungen oder sonstige strukturierte Maßnahmen zur Prävention von berufsbedingten Hauterkrankungen?
☐ Ja, regelmäßig ☐ Ja, ab und zu ☐ Nein

Eigene Einschätzung

Verschlechtert sich der Hautzustand Ihrer Hände durch die Tätigkeit?
☐ Nein ☐ Ja, folgende:

Werden Ihre Hautveränderungen während des Urlaubs besser?
☐ Überhaupt nicht ☐ Leicht ☐ Mäßig ☐ Deutlich

Wie stark haben Ihre Hautveränderungen bzw. Symptome Ihre **Arbeit** in den letzten 12 Monaten beeinträchtigt?
0 (überhaupt nicht beeinflusst) ☐ ☐ ☐ ☐ ☐ ☐ ☐ ☐ ☐ ☐ ☐ 10 (extrem beeinflusst) ☐ keine Angabe

MUSTER

Eigene Einschätzung [Fortsetzung]

Wie stark haben Ihre Hautveränderungen bzw. Symptome Ihren **Haushalt** in den letzten 12 Monaten beeinträchtigt?	0 (überhaupt nicht beeinflusst)	☐ ☐ ☐ ☐ ☐ ☐ ☐ ☐ ☐ ☐ ☐	10 (extrem beeinflusst)	☐ keine Angabe
Wie stark haben Ihre Hautveränderungen bzw. Symptome Ihren **Sport** in den letzten 12 Monaten beeinträchtigt?	0 (überhaupt nicht beeinflusst)	☐ ☐ ☐ ☐ ☐ ☐ ☐ ☐ ☐ ☐ ☐	10 (extrem beeinflusst)	☐ keine Angabe
Wie stark haben Ihre Hautveränderungen bzw. Symptome Ihre **Freizeit** (außer Sport) in den letzten 12 Monaten beeinträchtigt?	0 (überhaupt nicht beeinflusst)	☐ ☐ ☐ ☐ ☐ ☐ ☐ ☐ ☐ ☐ ☐	10 (extrem beeinflusst)	☐ keine Angabe

Welche Fragen stellen Sie sich bezüglich beruflicher Hauterkrankungen und Hautschutz? Welche Themengebiete interessieren Sie? *[Wir würden uns über eine Antwort und die damit verbundene Anregung sehr freuen!]*

MUSTER
