# Supplementary material for: Hand eczema symptoms, exposures and skin care in orthodontics: A national, cross-sectional questionnaire-based survey
Source: J Orofac Orthop. 2024 Apr 3;86(5):327–36. doi: 10.1007/s00056-024-00524-3 (PMC12373531; doi:10.1007/s00056-024-00524-3)
Supplement: Supplementary file 2 — Supplementary Tables 1–3 [file 56_2024_524_MOESM2_ESM.pdf]

| Skin experienced stress outside primary occupation (%) | n = 207, (100%) |
|---|---|
| No | 148 (71.5%) |
| Yes | 59 (28.5%) |
| History of rhinitis or asthma | n = 208, (99.5%) |
| No | 116 (55.8%) |
| Yes | 92 (44.2%) |
| ↳Time of eczema diagnosis | n = 45, (21.5%) |
| More than 12 months ago | 26 (57.8%) |
| In the last 3 months | 2 (4.4%) |
| In the last 3-12 months | 5 (11.1%) |
| Current | 12 (26.7%) |

**Supplementary Table 1: Eczema-focused medical history.** Percentages of responses to questions summarized in headings are highlighted in grey in relation to the overall respondents and the corresponding subgroups (n)

| | |
|---|---|
| Seasonal dependency | n = 209, (100%) |
| No | 159 (76.1%) |
| Yes | 50 (23.9%) |
| Exacerbation in spring | n = 209, (100%) |
| No | 196 (93.8%) |
| Yes | 13 (6.2%) |
| Exacerbation in summer | n = 209, (100%) |
| No | 200 (95.7%) |
| Yes | 9 (4.3%) |
| Exacerbation in autumn | n = 209, (100%) |
| No | 187 (89.5%) |
| Yes | 22 (10.5%) |
| Exacerbation through materials, chemicals, or other factors | n = 196, (93.8%) |
| No | 99 (50.5%) |
| Unclear | 23 (11.7%) |
| Yes | 74 (37.8%) |
| ↳ Exacerbation through gloves at work | n = 110, (52.6%) |
| No | 69 (62.7%) |
| Yes | 41 (37.3%) |
| ↳ Exacerbation through detergents at work | n = 110, (52.6%) |
| No | 59 (53.6%) |
| Yes | 51 (46.4%) |
| ↳ Exacerbation through solvents at work | n = 110, (52.6%) |
| No | 93 (84.5%) |
| Yes | 17 (15.5%) |
| ↳ Exacerbation through disinfectants at work | n = 110, (52.6%) |
| No | 45 (40.9%) |
| Yes | 65 (59.1%) |
| ↳ Exacerbation through frequent hand washing at work | n = 110, (52.6%) |
| No | 41 (37.3%) |
| Yes | 69 (62.7%) |
| ↳ Exacerbation through work with moist or wet hands at work | n = 110, (52.6%) |
| No | 75 (68.2%) |
| Yes | 35 (31.8%) |
| ↳ Exacerbation through stress at work | n = 110, (52.6%) |
| No | 78 (70.9%) |
| Yes | 32 (29.1%) |

| | |
|---|---|
| ↳ Exacerbation through food preparation at work | n = 110, (52.6%) |
| No | 109 (99.1%) |
| Yes | 1 (0.9%) |
| ↳ Exacerbation through oils at work | n = 110, (52.6%) |
| No | 110 (100%) |
| Yes | 0 (0.0%) |
| ↳ Exacerbation through paints at work | n = 110, (52.6%) |
| No | 108 (98.2%) |
| Yes | 2 (1.8%) |
| ↳ Exacerbation through adhesives at work | n = 110, (52.6%) |
| No | 102 (92.7%) |
| Yes | 8 (7.3%) |
| ↳ Exacerbation through dust at work | n = 110, (52.6%) |
| No | 100 (90.9%) |
| Yes | 10 (9.1%) |
| ↳ Exacerbation through waste or garbage at work | n = 110, (52.6%) |
| No | 108 (98.2%) |
| Yes | 2 (1.8%) |
| ↳ Exacerbation through other factors at work | n = 110, (52.6%) |
| No | 99 (90.0%) |
| Yes | 11 (10.0%) |
| ↳ Exacerbation through gloves during leisure time | n = 110, (52.6%) |
| No | 108 (98.2%) |
| Yes | 2 (1.8%) |
| ↳ Exacerbation through detergents during leisure time | n = 110, (52.6%) |
| No | 64 (58.2%) |
| Yes | 46 (41.8%) |
| ↳ Exacerbation through solvents during leisure time | n = 110, (52.6%) |
| No | 101 (91.8%) |
| Yes | 9 (8.2%) |
| ↳ Exacerbation through disinfectants during leisure time | n = 110, (52.6%) |
| No | 90 (81.8%) |
| Yes | 20 (18.2%) |
| ↳ Exacerbation through frequent hand washing during leisure time | n = 110, (52.6%) |
| No | 63 (57.3%) |
| Yes | 47 (42.7%) |
| ↳ Exacerbation through work with moist or wet hands during leisure time | n = 110, (52.6%) |

| | |
|---|---|
| No | 84 (76.4%) |
| Yes | 26 (23.6%) |
| ↳ Exacerbation through stress during leisure time | n = 110, (52.6%) |
| No | 91 (82.7%) |
| Yes | 19 (17.3%) |
| ↳ Exacerbation through food preparation during leisure time | n = 110, (52.6%) |
| No | 102 (92.7%) |
| Yes | 8 (7.3%) |
| ↳ Exacerbation through oils during leisure time | n = 110, (52.6%) |
| No | 108 (98.2%) |
| Yes | 2 (1.8%) |
| ↳ Exacerbation through paints during leisure time | n = 110, (52.6%) |
| No | 107 (97.3%) |
| Yes | 3 (2.7%) |
| ↳ Exacerbation through glue during leisure time | n = 110, (52.6%) |
| No | 108 (98.2%) |
| Yes | 2 (1.8%) |
| ↳ Exacerbation through dust during leisure time | n = 110, (52.6%) |
| No | 101 (91.8%) |
| Yes | 9 (8.2%) |
| ↳ Exacerbation through waste or garbage during leisure time | n = 110, (52.6%) |
| No | 106 (96.4%) |
| Yes | 4 (3.6%) |
| ↳ Exacerbation through other factors during leisure time | n = 110, (52.6%) |
| No | 104 (94.5%) |
| Yes | 6 (5.5%) |
| ↳Time lapse until exacerbation after contact (hours) | n = 39, (18.7%) |
| Mean (SD) | 3.0 (4.2) |
| Median (IQR) | 1.0 (2.0) |

**Supplementary Table 2: Exacerbation factors.** Percentages of responses to questions summarized in headings are highlighted in grey in relation to the overall respondents and the corresponding subgroups (n). (↳) indicates the proportion for the grey highlighted topic of the previously mentioned corresponding subgroup

| Material of work gloves | (multiple answers possible) |
|---|---:|
| Latex, natural rubber | 91 (43.5%) |
| Synthetic rubber | 134 (64.1%) |
| Plastic | 7 (3.3%) |
| Leather | 1 (0.5%) |
| Unknown material | 1 (0.5%) |
| Skin problems affect work [NRS10] | n = 186, (89.0%) |
| Mean (SD) | 1.1 (2.0) |
| Median (IQR) | 0 (2.0) |
| Skin problems affect household [NRS10] | n = 183, (87.6%) |
| Mean (SD) | 1.1 (1.8) |
| Median (IQR) | 0 (2.0) |
| Skin problems affect sports [NRS10] | n = 183, (87.6%) |
| Mean (SD) | 0.5 (1.3) |
| Median (IQR) | 0.0 (0.0) |
| Skin problems affect leisure time [NRS10] | n = 181, (86.6%) |
| Mean (SD) | 0.6 (1.4) |
| Median (IQR) | 0.0 (1.0) |

**Supplementary Table 3: Workplace factors and impairment in daily activities.** Percentages of responses to questions summarized in headings are highlighted in grey in relation to the overall respondents and the corresponding subgroups (n)
